# Terrorist Attacks against Sports Venues: Emerging Trends and Characteristics Spanning 50 Years

**DOI:** 10.1017/S1049023X23000377

**Published:** 2023-06

**Authors:** Grace R. Rahman, Gregory N. Jasani, Stephen Y. Liang

**Affiliations:** 1. University of North Carolina Gillings School of Global Public Health, Chapel Hill, North Carolina USA; 2. Department of Emergency Medicine, University of Maryland School of Medicine, Baltimore, Maryland USA; 3. Department of Emergency Medicine, Washington University School of Medicine, St. Louis, Missouri USA; 4. Division of Infectious Diseases, Department of Medicine, Washington University School of Medicine, St. Louis, Missouri USA

**Keywords:** disaster medicine, emergency preparedness, soccer, stadium, terrorism

## Abstract

**Introduction:**

Sports venues foster community and support local economies. Due to their capacity to host hundreds to thousands of spectators, sports venues are vulnerable to becoming targets of terrorism. Types of venues targeted, regional trends, and methods of attack employed world-wide have not been well-described.

**Methods:**

A search of the Global Terrorism Database (GTD) was conducted from 1970 through the end of 2019. Pre-coded variables for target type “business” and target subtype “entertainment/cultural/stadium/casino” were used to identify attacks involving venues where sports events might be viewed by spectators as part of an audience. Sports venues were specifically identified using the search terms “sport,” “stadium,” ”arena,” and “ring,” as well as mention of any specific sport. Two authors then manually reviewed each entry for specific information to confirm appropriateness for inclusion, selecting preferentially for attacks against venues where watching a sports event was the primary focus for the majority of the attendees. Descriptive statistics were performed using R (3.6.1).

**Results:**

Seventy-four (74) terrorist attacks targeting sports venues were identified from January 1, 1970 through December 31, 2019. Thirty-three (33) attacks, or 44.6% of attacks, involved soccer stadiums or soccer venues, while 33.8% of attacks (25 attacks) involved unspecified sports venues. A bombing or explosion was the most frequent method of attack employed, comprising 87.8% of attacks. The highest number of attacks occurred in the Middle East & North Africa. In total, 213 persons died and 699 more were wounded in attacks against sports venues.

**Conclusion:**

Although terrorist attacks against sports venues are uncommon, they carry the risk of mass casualties, especially when explosives are used. A greater understanding of the threat posed by terrorist attacks against sports venues can aid emergency preparedness planning and future medical responses.

## Introduction

Sports venues promote community through the common experience of an athletic competition. They are also an integral part of local economies. Venues for watching sports events can include stadiums, arenas, racetracks, and other large spaces. Due to their capacity to host hundreds to thousands of spectators at a time, as well as the media attention that many professional sports organizations receive, sports venues can be potential targets for terrorism.

Terrorist attacks against sports venue have not been well-described apart from individual high-profile events such as the 2015 attack against the Stade de France in Paris.^1^ Assessing the threat of terrorist attack against sports venues and better understanding the injuries likely to be encountered among victims can aid emergency preparedness planning and inform medical responses.

In this retrospective study, the Global Terrorism Database (GTD) was analyzed to determine the frequency of attacks against sports venues, the methods of attack used, and the extent of the casualties incurred over the past 50 years.

## Methods

A structured database search of the GTD was performed. The GTD is an open-source database maintained by the National Consortium for the Study of Terrorism and Responses to Terrorism (START; College Park, Maryland USA) reporting information on terrorist attacks occurring from 1970 through 2020.^2^ To be included in the GTD, an incident must be intentional, entail some level of violence or immediate threat of violence, and be perpetrated by sub-national actors. Furthermore, an incident must meet at least two of the following three criteria: (1) the act must be aimed at attaining a political, economic, religious, or social goal; (2) there must be evidence of an intention to coerce, intimidate, or convey some other message to a larger audience than the immediate victims; and/or (3) the action must be outside the context of legitimate warfare activities.^2^

Pre-coded variables for target type “business” and target subtype “entertainment/cultural/stadium/casino” were used to identify attacks in the GTD involving venues where sports events might be viewed by spectators as part of an audience. Sports venues were identified using the search terms “sport,” “stadium,” ”arena,” and “ring,” as well as for any specific mention of a type of sport by one of the authors (SYL). Two authors (GRR & SYL) manually reviewed each entry, including free-text variables, for specific target information to confirm appropriateness for inclusion in the analysis, selecting preferentially for attacks against venues where watching a sports event was the primary focus for the majority of attendees. If consensus could not be reached regarding inclusion or exclusion of an entry, a third author (GNJ) reviewed the entry to resolve any discrepancy. Stadiums, sports complex, and other venues where the primary focus was not to watch a sports event were excluded. Variables analyzed included date of incident, country of incident, method of attack, and number of victims killed or wounded. A new variable classifying the type of sports venue was created based on specific target information provided in the free-text variable mentioned before.

Data were analyzed using R (version 3.6.1; R Foundation for Statistical Computing; Vienna, Austria).^3^ Categorical variables were reported using frequencies [n (%)] and continuous variables using median and interquartile range (IQR). This study was determined to be exempt from review by the Washington University School of Medicine (St. Louis, Missouri USA) Human Research Protection Office.

## Results

**Figure 1.**
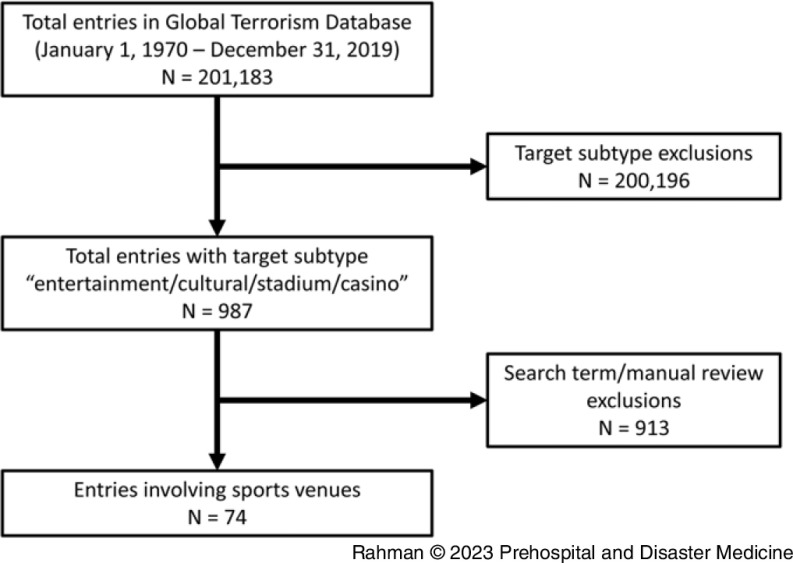
Flow Diagram for Inclusion/Exclusion of Entries from the Global Terrorism Database.

A total of 201,183 entries involving intentional global incidents were reported in the GTD from January 1, 1970 through December 31, 2019. Of those, 987 entries were identified by the pre-coded subtype variable for “entertainment/culture/stadium/casino.” Upon further review, 74 terrorist attacks targeting sports venues were included in the analysis (Figure 1).

**Figure 2.**
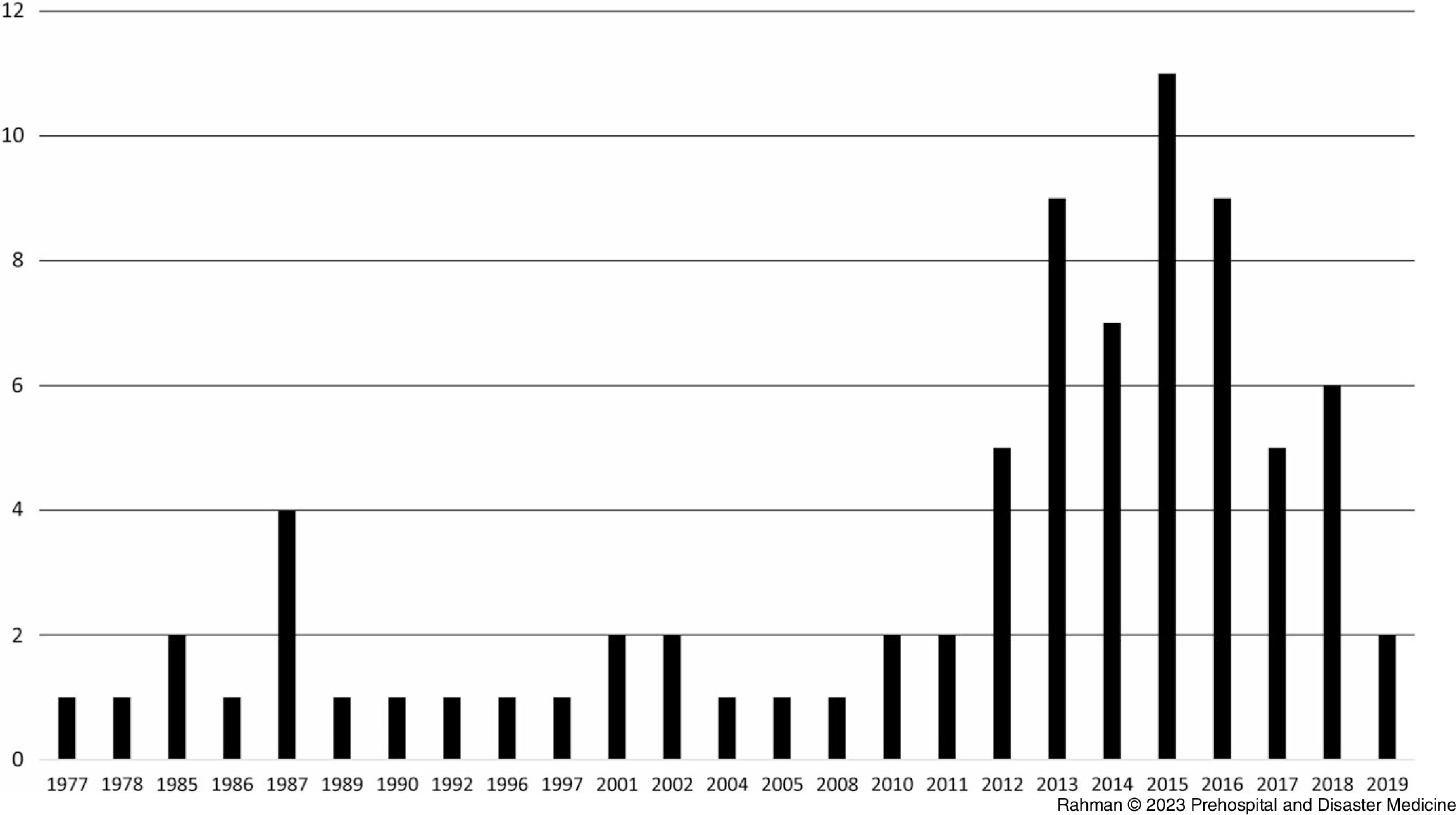
Number of Terrorist Attacks per Year against Sports Venues.

The greatest number of attacks against sports venues in any one year were 11 in 2015 and nine in 2016 (Figure 2). Thirty-three (33) attacks (44.6%) involved soccer stadiums or other soccer venues (Table 1). Twenty-five (25) attacks (33.8%) involved unspecified sports venues without further detail regarding the sport played at the time of the attack. Nine attacks (12.1%) involved other sports, such as boxing, bullfighting, hockey, horse racing, and wrestling. Seven attacks (9.5%) involved cricket stadiums. The highest number of attacks occurred in the Middle East & North Africa, specifically Iraq (25). Attacks on sports venues were carried out in 26 different countries.

**Table 1.** Characteristics of Terrorist Attacks against Sports Venues

Characteristics, no. (%)	
Sports Venue Type (n = 74)	
Soccer	33 (44.6)
Sports Venue, Unspecified	25 (33.8)
Other Sports^a^	9 (12.1)
Cricket	7 (9.5)
Region	
Middle East & North Africa	36 (48.6)
South Asia	13 (17.6)
Western Europe	7 (9.5)
South America	7 (9.5)
Eastern Europe	4 (5.4)
Sub-Saharan Africa	3 (4.1)
North America	2 (2.7)
East Asia	1 (1.3)
South Asia	1 (1.3)
Attack Type	
Bombing/Explosion	65 (87.8)
Facility/Infrastructure Attack	3 (4.1)
Armed Assault	3 (4.1)
Hostage Taking (Kidnapping)	2 (2.7)
Unknown	1 (1.3)
Weapon Type Used in Attack	
Explosives	66 (89.2)
Firearms	3 (4.1)
Incendiary	3 (4.1)
Unknown	2 (2.7)
Suicide Attack	7 (9.5)
Victims, number [median (IQR)]	
Killed, Total	213 [0 (0-48)]
Soccer	113 [1 (0-31)]
Sports Venue, Unspecified	75 [0 (0-48)]
Other Sports^a^	17 [0 (0-160)]
Cricket	8 [0 (0-4)]
Wounded, Total	699 [0.5 (0-164)]
Soccer	350 [4 (0-102)]
Sports Venue, Unspecified	259 [1 (0-164)]
Other Sports^a^	81 [0 (0-51)]
Cricket	38 [4 (0-23)]

a Other sports: boxing, bullfight, hockey, horse racing, and wrestling.

A bombing or explosion was the most frequent type of attack, occurring in 65 (87.8%) attacks, followed by damage to a facility or infrastructure (excluding the use of an explosive) in three (4.1%) attacks, armed assault in three (4.1%) attacks, and hostage-taking in two (2.7%) attacks (Table 1). Perpetrators used explosives in 66 (89.2%) attacks, firearms in three (4.1%) attacks, and incendiary weapons in three (4.1%) attacks. Two attacks were executed using an unknown weapon. Upon further stratification, explosives were the most common weapon type employed across all types of sports venues identified, including 31 (93.9%) soccer venues, 23 (92.0%) unspecified sports venues, five (55.6%) other sports venues, and seven (100.0%) cricket venues.

In total, 213 persons (median = 0; IQR: 0-48) died and 699 persons (median = 0.5; IQR: 0-164) were wounded in attacks against sports venues from 1970 through the end of 2019 (Table 1). Attacks involving soccer venues contributed the greatest number of casualties with 113 deaths and 350 wounded.

## Discussion

Sports venues elevate athletic competition to a shared experience by a large audience and are important fixtures in many communities. They also support local economies and create jobs. Unfortunately, sports venues have also been the targets of terrorism. This study sought to analyze trends from prior attacks in the hopes of informing future emergency preparedness and medical responses.

At least 74 terrorist attacks against sports venues have been documented in the GTD. While the venues targeted represent a range of sports, soccer stadiums have been targeted most frequently, accounting for nearly 45% of all attacks. Admittedly, one-third of the attacks identified did not specify the type of sports venue targeted. However, the frequency with which soccer stadiums have been impacted likely speaks to the global popularity of soccer as well as the ability of the sport to attract large audiences. Prior to 2011, the most attacks seen in one year was four in 1987. Since 2011, each subsequent year has consistently seen over four attacks, with a peak of 11 in 2015 (Figure 2). Not surprisingly, bombings and explosions are the most frequent method of attack utilized. Given that most sports venues assemble spectators together in close proximity to one another, bombings have the potential to inflict numerous casualties rapidly. Bombs and explosives can also be portable and concealable and can be inexpensive to manufacture.^4^

Terrorist attacks against sports venues have the potential to strain or even overwhelm the health care system; hospitals should have a plan to manage sudden surges in patients with polytrauma.^5^ During the complex coordinated terrorist attacks of November 2015 in Paris, assailants detonated three explosives at the Stade de France. The first detonation occurred outside the stadium. A second detonation occurred when a suicide bomber was discovered to be wearing an explosive vest by personnel at a security checkpoint prior to entering the stadium.^6^ The third detonation occurred outside a separate stadium entrance. In all, 53 people were injured at the Stade de France. Several additional attacks occurred elsewhere throughout Paris over the ensuing hours. Developed over 20 years ago for such an event, Paris’ emergency response plan, the “White Plan,” mobilized all 40 Paris hospitals, including “100,000 health professionals, 22,000 beds, and 200 operating rooms.”^1,7^ The “White Plan” mitigated the surge in patients and provided medical care to all those affected by the attacks.^1,8^ Additionally, the plan initiated a psychological support center with 35 psychiatrists along with psychologists, nurses, and volunteers to provide mental health care to patients, family members, and medical professionals participating in the response.^7^ All too often after an attack, the immediate focus is on the physical well-being of the victims; the negative mental health impacts of the event are frequently overlooked and can linger long after physical wounds have healed.^9^ Over the course of the Paris attacks, 124 people died, 643 were injured and received medical care, seven more died in the hospital, and over 1,500 people received mental health care.^8^ Paris’ highly-organized emergency response highlights the importance of multidisciplinary collaboration and detailed, proactive planning for mass-casualty events.^7^

Medical emergency preparedness planning should start with increasing situational awareness. Health care facilities near large sports venues should incorporate event schedules into all-hazards planning for receiving mass casualties. Regular training and exercises can help familiarize emergency clinicians with hospital protocols for triage and management of the wide spectrum of injuries possible in a mass-casualty event.^4^ Medical directors for Emergency Medical Services (EMS) and other prehospital clinicians should collaborate with sports venues in emergency planning. Formal designation of a law enforcement medical coordinator to serve as a liaison between EMS, fire/rescue, and police can enhance communication during a response, optimize use of prehospital medical assets and local hospital resources, and provide medical expertise to incident commanders in high-stress situations.^4^

While not specific to terrorist attacks, emergency preparedness planning should recognize and mitigate the historical risk of stampedes at sports venues. Stampedes occur when crowd surges result in trampling of individuals with subsequent crush-related injuries. In particular, stampedes can result in traumatic asphyxia and death of individuals compressed or trampled over.^10^ Stampedes may result from an over-excited crowd rushing the playing field to celebrate a victory or conversely seeking escape from a threat. In one such event in Johannesburg, South Africa, tear gas was deployed during a soccer match in 2001 to disperse overcrowding, but led to a stampede resulting in 43 deaths and 250 injured.^11^ More recently in October 2022, a stampede of agitated spectators dispersed with tear gas following a soccer match loss at an Indonesian stadium led to 130 deaths.^12^ The management of traumatic asphyxia relies on timely airway and respiratory support; in the event of mass casualties with this injury, first responders and emergency clinicians may be significantly engaged in individual care, including cardiopulmonary resuscitation. Crowd management plans should mitigate surges, promote safe egress of spectators, and facilitate on-site availability or rapid entry of first responders to care for the injured, all while maintaining the security of the venue.^10^

## Limitations

The strengths of this study include the size and scope of the GTD, considered the most comprehensive, unclassified database of terrorist attacks world-wide available to researchers. However, source data for the GTD originates from publicly available materials including media reports, as well as existing datasets, secondary sources (eg, books and journals), and legal documents, but not medical records, and is based on convenience sampling. Additionally, the GTD does not include foiled or failed plots, attacks in which violence is threatened as a means of coercion, incidents reported from non-high-quality sources, or attacks in conflict zones where the combatant may be “national” and fall out of their inclusion criteria. Access to reliable source materials and efficiency of workflows has varied over the long history of the GTD. Data validation is accomplished through inclusion of only high-quality sources, as defined by START.^2^ All these limitations may mean that the true incidence and human toll of terrorist attacks targeting sports venues could be under-reported or misreported. It is also possible that the data under-report the number of individuals killed or wounded; in many instances, these numbers are not known and so no numbers are reported. Finally, attacks where terrorism is a strong possibility, but some uncertainty exists, are included in the GTD and identified as such for incidents occurring after 1997. Of the 74 attacks included in this analysis, 25 (35.1%) involved unspecified sports venues where the type of sport played was not described, which could have led to under-estimation of attacks attributed to sports identified in this analysis, including soccer.

## Conclusion

Although terrorist attacks against sports venues are not common, they carry a risk of mass casualties, especially when explosives are used. A greater understanding of the threat posed by terrorist attacks against sports venues can aid emergency preparedness planning and future medical responses.
